# The Effects of Vitamin D Supplementation on Metabolic and Oxidative Stress Markers in Patients With Type 2 Diabetes: A 6-Month Follow Up Randomized Controlled Study

**DOI:** 10.3389/fendo.2021.610893

**Published:** 2021-08-19

**Authors:** Milena Cojic, Radivoj Kocic, Aleksandra Klisic, Gordana Kocic

**Affiliations:** ^1^Primary Health Care Center, Faculty of Medicine, University of Montenegro, Podgorica, Montenegro; ^2^Clinic for Endocrinology, Faculty of Medicine, University of Nis, Nis, Serbia; ^3^Institute of Biochemistry, Faculty of Medicine, University of Nis, Nis, Serbia

**Keywords:** vitamin D supplementation, HbA1c, insulin resistance, oxidative stress, type 2 diabetes

## Abstract

Vitamin D deficiency could play an important role in the pathogenesis of type 2 diabetes mellitus (T2DM) as it may alter several crucial processes in the development of diabetes and its complications, such as pancreatic insulin secretion, peripheral insulin resistance, persistence of systemic „sterile” inflammation and immune activation. Vitamin D may also have an antioxidant effect through the inhibition of free radicals generation. The reported study was designed with eligible consecutively recruited patients with T2DM on standard metformin therapy (n=130), randomized in 1:1 ratio, considered to have undergone Vitamin D supplementation according to the guidelines proposed by the Endocrine Society, or to have continued with metformin only. The potential benefit was monitored through the influence on glycemia level, glycated haemoglobin (HbA1c), insulin resistance index (calculated as homeostatic model assessment; HOMA-IR), Castelli Risk Index I and Tryglicerides/Thiobarbituric acid*-*reactive substances (TG/TBARS) Index in a 6-month follow up period. Our study indicates that oral daily doses of vitamin D improve HbA1c levels over the 3-month and 6-month period, followed by a significant decrease in advanced oxidation protein products levels over the 3-month period when higher vitamin D doses are given. The effect of vitamin D on HOMA-IR index, malondialdehyde levels and TG/TBARS index was not statistically significant. Further investigation should consider defining the doses of vitamin D in patients with T2DM which may attenuate the oxidative stress risk, the risk of metabolic syndrome and the risk of related cardiovascular events.

## Introduction

During the past years there has been a growing interest in extra-skeletal effects of vitamin D since it has been discovered that vitamin D receptors are expressed not only in tissues related to bone metabolism, but also in many other tissues, such as brain, prostate, breasts, immune cells etc. (1, 2). This has linked vitamin D to many chronic diseases, such as cancer, heart disease, metabolic syndrome, prediabetes, diabetes, inflammatory and autoimmune diseases (3, 4).

Patients with type 2 diabetes mellitus (T2DM) usually have a lack of vitamin D but it is still unknown whether this is a coincidence or whether low levels of vitamin D may contribute to the disease appearance. Recent studies have suggested that vitamin D deficiency could play an important role in T2DM pathogenesis through altering several crucial processes in the development of diabetes and its complications: pancreatic insulin secretion, peripheral insulin resistance, down-regulation of the insulin receptor gene, systemic „sterile” inflammation and immune activation (3, 5). Besides hyperinsulinemia and hyperglycaemia, T2DM is a condition characterized by an increased formation of free radicals and a decreased antioxidant capacity. Some experimental studies have shown that vitamin D may have an antioxidant effect followed by the inhibition of free radicals generation, consequent lipid peroxidation and oxidative modification of other biomolecules (6). Due to the abovementioned effects, vitamin D supplementation has been proposed as a possible therapeutic tool for T2DM to optimize glycaemic control and to prevent the occurrence of complications (7).

Despite the promising data from observational studies, vitamin D supplementation trials have yielded inconsistent results. Some of them have reported a significant reduction of fasting blood glucose (FBG) and glycated haemoglobin (HbA1c) while others have shown no statistically significant improvement in vitamin D group vs. placebo (7–10). These results should be interpreted with caution because most of the trials included small heterogeneous groups of patients in different stages of disease with suboptimal doses of vitamin D, various ways of administration, lifestyle habits, dietary habits, age, ethnicity, season of the year, location and climate conditions (5, 7).

Moreover, little or no research has determined any long-term effect of vitamin D supplementation in T2DM patients with co-administration of the standard pharmacological regimen, by using daily doses of vitamin D according to the available guidelines (11). Therefore, the study reported was aimed at determining whether vitamin D supplementation in T2DM patients, who underwent standard metformin therapy, may have a benefit through the influence on insulin resistance, Castelli risk index I and oxidative stress markers, in a 6-month follow up randomized controlled study.

## Patients and Methods

### Study Design and Participants

A prospective, randomized, controlled open-label study was conducted in a summer-winter period (overall trial start date 02.04.2018; overall trial end date 15.12.2018.) in Primary Health Care Center Podgorica, Montenegro. The study was conducted according to the Declaration of Helsinki and Good clinical Practice guidelines. Ethical approval was obtained by the Ethical Committee of Primary Health Care Center in Podgorica (Ethical Committee of Primary Health Care Center in Podgorica, ID number 05/01-E.K.-5989/1) and all participants provided a written informed consent. Study was also registered on ISRCTN registry platform (02.12.2019. ID number ISRCTN25609316). In the region, Mediterranean climate prevails, at latitude 42°, where regular diet is characterized by Mediterranean eating habits, which may reflect a basal vitamin D level.

A sample size was determined according to the key variable HbA1c of previous study (12) considering α = 0.05 and statistical power 1-β =0.8. The calculated sample size was 49 patients per group. Out of 560 pre-screened T2DM patients, 150 met the eligibility criteria. Allowing the drop-out rate of 30%, a total of 130 participants (65 in each group) were enrolled, ≥ 30 years of age, diagnosed with T2DM according to American Diabetes Association (ADA) 2011 criteria (13), who had good metabolic control (HbA1c ≤ 7%), who were treated with metformin and were given lifestyle advice.

The exclusion criteria were as follows: the use of vitamin D supplements and any diabetes pharmacotherapy other than metformin 6 months prior the study; the use of drugs that affect the metabolism of vitamin D; the presence of severe anemia; a chronic liver or kidney failure; alcoholism, pregnancy; malabsorption; urolithiasis; hypercalcemia; body mass index (BMI) ≥ 40 kg/m2 and the presence of acute or chronic inflammatory conditions.

### Outcomes

The primary outcome of the study was change in insulin resistance and glycemic control measured by the homeostasis model of assessments (HOMA-IR) and glycemic parameters (FBG, HbA1c). The oxidative stress parametres [malondialdehyde (MDA) and advanced oxidation protein products (AOPP)] and inflammation markers [C-reactive protein (CRP)] were considered secondary outcomes. Furthermore, alteration of vitamin D levels over time, blood pressure, lipid profile, BMI, calcium total (Ca), calcium ionized (Ca ++) and atherogenic risk were also considered as secondary outcomes.

### Intervention

**Figure 1:**
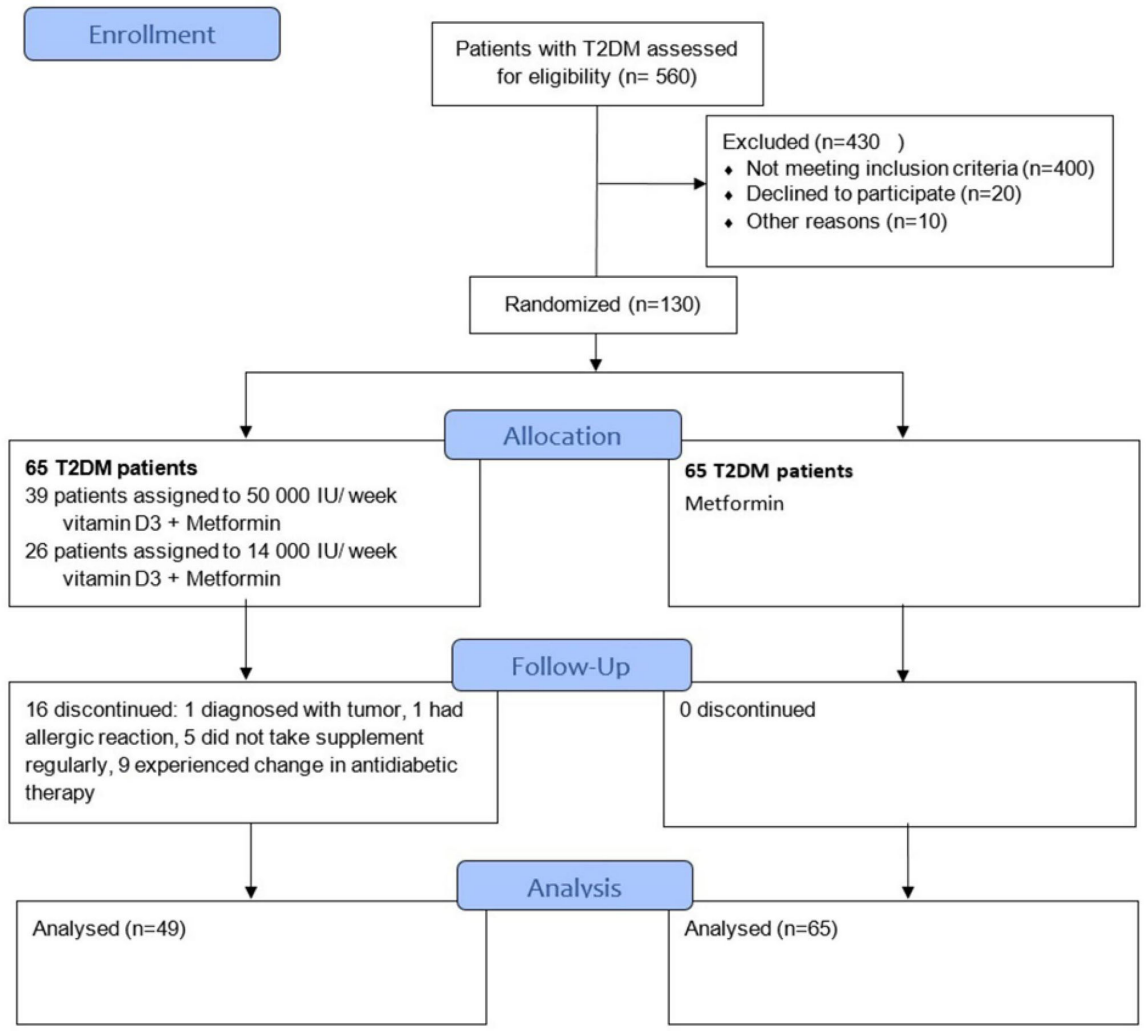
Participant flowchart and study design.

The eligible participants recruited consecutively were randomly assigned to two groups in 1:1 ratio. The Serum level of 25(OH)D (marker of vitamin D status) was measured at baseline in all participant*s.* Half of the patients (n=65) were randomly prescribed vitamin D3 therapy and continued their prescribed metformin therapy for 6 months, while the other group (n=65) continued their prescribed metformin therapy only. The dosing was carried out according to the Endocrine Society’s clinical practice guidelines as for the vitamin D baseline levels (10). The supplements were given in the form of Vigantol oil drops (Merck KGaA, 0.5 mg/ml, one drop equals 500 IU). The participants in the first group who were vitamin D deficient [defined as serum levels of 25(OH)D ≤ 50 nmol/L] were asked to take 50 000 IU of vitamin D3 weekly (equal to 15 drops of vitamin D supplement-Vigantol oil daily) during the first 3 months and 14 000 IU weekly (4 drops daily) for the next 3 months. The participants in the same group whose 25(OH)D levels were > 50 nmol/L were asked to take 14 000 IU weekly (4 drops daily) until the end of the study. The study protocol is shown in **Figure 1**. At the screening visit, data about participant’s level of physical activity, sun exposure and average dietary intake of vitamin D of three consecutive days were also reported. Every participant received a table with a list of food containing vitamin D and was advised not to alter the usual dietary amounts of vitamin D. All participants also agreed not to change physical activity and sun exposure patterns while enrolled. The compliance was carefully controlled by the medical staff. The Patients whose diabetes therapy has changed during the study period were excluded. The report involved clinical and laboratory analyses of data through the 3- and the 6-month assessment period. All supplements were provided to the patients by the authors themselves through the medical staff. Supplement adherence was assessed at the end of every month by returning the empty bottles of Vigantol oil. In the Metformin + Vitamin D group, a total of 16 patients were excluded (Six with assigned regimen of 50 000 IU+14 000 IU, and 10 with assigned regimen of 14 000 IU): 1 patient was diagnosed with pancreatic tumor, 1 developed urticaria-like skin lesions after taking first dose of supplement and reaction was classified as moderate, 5 patients did not take the supplement regularly and 9 patients changed diabetes medication. In the Metformin group all 65 patients completed the study successfully.

### Follow Up and Outcome Measurements

All participants were subjected to anthropometric measurements and laboratory tests at baseline, 3 and 6 months of intervention. Body height in centimeter*s* was measured without shoes using a wall-mounted stadiometer. Body weight was measured in wearing light clothes and without shoes on electronic (digital reading) scales previously calibrated with a possible error of *±* 100g. BMI was calculated as kg/m^2^. Waist circumference (WC) was measured while standing and with clothes on using the non-stretchable tape positioned parallel to the floor over the umbilicus. Systolic (SBP) and diastolic blood pressure (DBP) were measured in a seated position after 10 minutes of rest with an electronic blood pressure monitoring device (Microlife’s BP A150-30 AFIB). Two measurements were done one minute apart and the average value was used.

### Blood Samples

Samples were drawn from venous blood early in the morning after 12 hours of overnight fasting. Serum levels of FBG, creatinine, total cholesterol (TC), low-density lipoprotein (LDL) cholesterol, high-density lipoprotein (HDL) cholesterol, triglycerides (TG) and Ca were measured using standard enzymatic procedures (Roche Cobas 6000 c 501, Mannheim, Germany). Ca ++ was determined using the ion-selective method on an electrolyte analyzer [Roche Diagnostics AVL 9180 Electrolyte Analyzer (AVL 9180, Roche, Japan)].

Serum CRP level was determined by immunoturbidimetric method (Roche Cobas 6000 c 501, Mannheim, Germany).

For HbA1c, blood was used with K2EDTA anticoagulants and it was determined immunoturbidimetrically (Roche Cobas 6000 c 501, Mannheim, Germany).

Fasting insulin (FI) and 25(OH)D serum levels were measured by electrochemiluminescence using commercial Roche tests on the Cobas 6000/e601 automated analyzer (Roche Diagnostics, Mannheim, Germany). This is a competitive electrochemiluminescence assay which employs a vitamin D binding protein as capture protein, for the quantitative determination of total 25-OH vitamin D in human serum and plasma. According to the manufacturer’s instruction measuring range is 3 to 70 ng/mL (7.5 - 175.0 nmol/L), functional sensitivity 4.01 ng/mL (10.0 nmol/L) (CV 18.5%), within-run precision: < 15 ng/mL: SD ≤ 1 ng/mL > 15 ng/mL: ≤ 6.5%, intermediate precision: < 15 ng/mL: SD ≤ 1.7 ng/mL > 15 ng/mL: ≤ 11.5%.

Lipid peroxidation was measured in plasma and expressed as the Thiobarbituric acid*-*reactive substances (TBARS) biomarker malondyadehide (MDA). The mean recovery was 90% (SD ± 2%), the CV was 4% (14). The TG/TBARS index was used to monitor the ratio between the potentially oxidizable substrates-polyunsaturated fatty acids of circulating triglycerides and their oxidized counterparts-lipid peroxides.

Advanced oxidation protein product level (AOPP) was measured in serum spectrophotometrically, expressed as chloramine-T equivalents. Before the analysis, plasma samples were diluted in 1:10 ratio (15). In order to avoid turbidity interference with lipids and drugs, plasma samples were previously treated with 2 mol/L magnesium chloride (MgCl_2_) and 4% phosphortungstate dissolved in 0.19 mol/L sodium hydroxide (NaOH), centrifuged at 1000*g* for 20 minutes, and supernatant was used for AOPP analyses (ubaci referencu).

Castelli risk index I was determined through the TC/HDL cholesterol ratio, reflecting cholesterol-related atherogenic risk, characteristic of the metabolic syndrome (16), recently studied as the potential surrogate marker related to the insufficiency of vitamin D and metabolic syndrome (17).

HOMA-IR was calculated by the formula HOMA−IR=FBG(mmol/L)×FI(μIU/L)/22.5 (18).

### Statistical Analysis

All normally distributed data were presented as mean ± SD. All data not normally distributed were presented as median and interquartile range. A Student’s t test was used to compare the continuous variables between the Metformin + Vitamin D and Metformin group, when the distribution was normal, and Mann-Whitney test when the data have not been distributed normally. Skewed continuous variables were natural log transformed before the analysis. Two way ANOVA for repeated measures was used to monitor parameters over time. Linear regression analysis was used to assess the mean difference between Metformin + Vitamin D and Metformin group after 3 and 6 months (the mean difference is reported as β). All effects were adjusted for gender, baseline age, BMI, FI and HbA1c. The significant p value was set as 0.05 (two-sided). All statistical analyses were performed using R software (version 3.4.3) (R Foundation for Statistical Computing, Vienna, Austria) (19).

## Results

Out of 560 pre-screened patients 130 consented to participate in the study. **Table 1** shows baseline demographic characteristics of the study population. Clinical and biochemical parameters were balanced between the groups.

**Table 1:** Baseline demographic characteristics of study population.

	Metformin + Vitamin D group N=49		Metformin group N=65		p-value
	N	%	N	%	
Age (years) 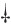	60.41 (8.5)		63.65 (8.2)		0.044^1^
Male 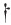	36	55.4	21	42.9	0.256^2^
T2DM duration (years) 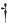	4 (5)		6 (5)		0.188^3^
Non smokers 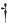	22	44.9	38	58.5	0.356^2^
Smokers 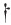	12	24.5	12	18.5	
Ex-smokers 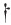	15	30.6	15	23.1	
Creatinine (μmol/L) 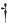	78 (21)		84 (24)		0.082^3^
Vitamin D (nmol/L) 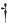	48.79 (31.63)		58.02 (32.32)		0.095^3^
FBG (mmol/L) 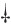	7.90 (1.4)		7.92 (1.5)		0.841^1^
HbA1c (%)	6.56 (1.02)		6.74 (0.81)		0.387^3^
FI (μU/L) 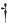	11.25 (7.43)		10.66 (8.92)		0.445^3^
HOMA-IR 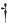	3.67 (2.63)		3.64 (3.22)		0.349^3^
BMI (kg/m2) 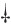	30.13 (4.6)		29.79 (5.0)		0.834^1^
Body weight (kg)	87.44 (14.1)		89.61 (18.5)		0.488^1^
Ca	2.43 (0.16)		2.48 (0.15)		0.462^1^
Ca ++	1.16 (0.09)		1.15 (0.07)		0.407^1^

Data are presented as 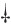 mean (SD) or 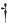 median (Interquartile range) or 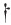 count, %. ^1^ t test, ^2^ Chi square test, ^3^ Mann-Whitney test. FBG, fasting blood glucose; HbA1c, glycated hemoglobin; FI, fasting insulin; HOMA-IR, Homeostatic Model Assessment of Insulin Resistance; BMI, Body mass inde;, Ca, total calcium; Ca ++, calcium ionized.

For vitamin D concentration, repeated measures two-way ANOVA revealed significant interaction (F=75.349, p<0.001), significant effect of group (F=37.976, p<0.001), and significant effect of time (F=115.201, p<0.001) (**Table 3**). In Metformin + Vitamin D group 25(OH)D levels increased significantly during intervention period (p<0.001). In Metformin group 25(OH)D levels significantly increased during the first 3-month period and then significantly decreased (p<0.001 for all) (**Table 2**). After 3 and after 6 months of supplementation, 25(OH)D levels differed significantly between groups (p<0.001) adjusted for participants’ age, gender and baseline levels of BMI, FI and HbA1c (**Table 4**).

**Table 2:** Comparison of measured parameters over the 6-month period of vitamin D treatment.

	Metformin + Vitamin D group			Metformin group		
	Baseline	3 month	6 month	Baseline	3 month	6 month
Vitamin D (nmol/L) 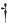	48.79 (31.63)^a,b^	104.70 (30.46)^b,#^	92.24 (20.25)^#^	58.02 (32.32)^,c,d,^	67.44 (25.13)^d^	51.77 (23.99)
HbA1c (%) 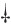	6.56 (1.02)^a^	6.32 (0.69)^b,#^	6.48 (0.70)^#^	6.74 (0.81)^c^	6.66 (0.85)^d^	6.87 (0.92)
FI (μU/L) 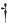	11.25 (7.43)	9.81 (7.04)	11.26 (6.68)	10.66 (8.92)	12.62 (8.30)	11.92 (7.86)
BMI (kg/m2) 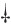	30.1 (4.6)	30.1 (4.6)	29.7 (7.8)	29.8 (5.0)	25.7 (10.9)	28.8 (6.1)
WC (cm)	103 (11)	103 (11)	104 (11)	105 (11)	106 (10)	105 (11)
HOMA-IR 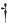	3.67 (2.63)	3.63 (3.02)	3.44 (2.89)	3.64 (3.22)	3.42 (2.64)	3.39 (3.37)
FBG (mmol/L) 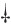	7.87 (2.4)^a,b^	7.28 (1.16)	7.23 (1.26)	7.91 (1.44)	7.97 (1.74)	7.74 (1.49)
SBP (mmHg) 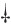	136.69 (24)^a^	142.48 (17.33)^b^	136.65 (17.78)	139.48 (19.15)	142.52 (17.33)	141.45 (16.62)
DBP (mmHg) 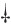	83.19 (12.14)	85.23 (9.03)	83.07 (8.21)	81.28 (10.16)	84.01 (9.91)	84.18 (9.67)
MDA (TBARS) (μM/L) 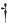	3.14 (1.89)	2.97 (1.21)	2.77 (2.38)	3.31 (1.73)^d^	3.17 (1.32)^d^	3.09 (3.97)
AOPP (µM chloramines T equivalents) 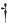	185.74 (146.48)^a^	131.19 (64.64)^b,#^	220.28 (63.73)	176.02 (134.35)^c^	104.56 (67.32)^d^	199.42 (78.28)
CRP (mg/L) 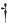	1.79 (3.02)	1.36 (2.55)	1.61 (2.93)	1.40 (2.06)	1.49 (2.08)	2.13 (3.16)
TC (mmol/L) 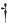	5.26 (1.92)^a^	5.10 (1.72)^b^	5.72 (1.60)	5.40 (1.64)	5.30 (1.14)	5.60 (2.01)
TG (mmol/L) 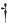	1.72 (1.02)	1.41 (0.99)	1.70 (1.08)	1.77 (1.04)	1.90 (1.24)	1.85 (1.40)
HDL (mmol/L) 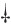	1.35 (0.34)	1.33 (0.31)^#^	1.38 (0.30)	1.26 (0.30)^c^	1.19 (0.26)^d,#^	1.25 (0.34)
LDL (mmol/L) 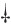	3.36 (1.06)	3.18 (0.98)	3.40 (1.04)	3.41 (1.05)	3.13 (0.90)	3.22 (1.19)
Castelli 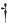	4.00 (2.07)	4.09 (1.75)	3.99 (1.67)	4.52 (2.03)	4.30 (1.57)	4.51 (1.70)
TG/TBARS 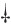	0.56 (0.32)	0.58 (0.32)	0.55 (0.28)	0.60 (0.30)	0.70 (0.32)	0.62 (0.34)
Ca	2.43 (0.16)	2.44 (0.16)	2.48 (0.15)	2.41 (0.10)	2.41 (0.11)	2.44 (0.09)
Ca ++	1.16 (0.09)	1.22 (0.08)	1.23 (0.10)	1.15 (0.07)	1.21 (0.06)	1.23 (0.18)

Data are presented as 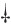 mean (SD) or 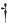 median (Interquartile range), In two way ANOVA for repeated measures: In the Metformin + Vitamin D group: p<0.05 ^a^vs 3 months, ^b^vs 6 months, In the Metformin: p<0.05 ^c^vs 3 months, ^d^vs 6 months, # between group at specific time point. HbA1c, glycated hemoglobin; FI, fasting insulin; BMI, Body mass index; WC, waist circumference; HOMA-IR, Homeostatic Model Assessment of Insulin Resistance; FBG, fasting blood glucose; SBP, systolic blood pressure; DBP, diastolic blood pressure; MDA, malondialdehyde; AOPP, advanced oxidation protein products; CRP, C-reactive protein; TC, total cholesterol; TG, triglycerides; HDL, high-density lipoprotein; LDL, low-density lipoprotein; TBARS, thiobarbituric acid reactive substance; Ca, total calcium; Ca ++, calcium ionized.

**Table 3:** Results of two-way ANOVA for effects of the Time factor and Group factor in the study.

Parameter	Source	F	p value	Partial Eta Squared
Vitamin D (nmol/L)	Time	115.201	<0.001	0.526
	Group	37.976	<0.001	0.267
	time * group	75.349	<0.001	0.420
HbA1c (%)	Time	9.782	<0.001	0.158
	group	3.999	0.048	0.037
	time * group	1.391	0.253	0.026
FI (μU/L)	Time	0.852	0.428	0.008
	Group	0.000	0.992	0.000
	time * group	0.389	0.628	0.008
BMI (kg/m2)	Time	1.479	0.231	0.016
	Group	0.231	0.632	0.003
	time * group	1.597	0.210	0.017
WC (cm)	Time	1.046	0.354	0.013
	Group	2.544	0.115	0.030
	time * group	0.860	0.418	0.010
HOMA-IR	Time	1.049	0.348	0.010
	Group	0.297	0.587	0.003
	time * group	0.694	0.491	0.006
FBG (mmol/L)	Time	3.978	0.020	0.037
	Group	2.304	0.132	0.022
	time * group	2.733	0.070	0.026
SBP (mmHg)	Time	3.233	0.046	0.039
	Group	0.488	0.487	0.006
	time * group	0.862	0.417	0.011
DBP (mmHg)	time	2.881	0.062	0.035
	Group	0.128	0.722	0.002
	time * group	1.264	0.285	0.016
MDA (TBARS) (μM/L)	Time	251.942	<0.001	0.716
	Group	0.021	0.884	0.000
	time * group	0.109	0.865	0.001
AOPP (chloramines T equivalents)	Time	53.710	<0.001	0.496
	Group	4.029	0.047	0.035
	time * group	0.187	0.667	0.002
CRP (mg/L)	Time	0.576	0.528	0.006
	Group	0.184	0.669	0.002
	time * group	1.260	0.282	0.012
TC (mmol/L)	Time	5.009	0.008	0.046
	Group	0.100	0.753	0.001
	time * group	0.355	0.694	.003
TG (mmol/L)	Time	0.342	0.673	0.003
	Group	2.610	0.109	0.025
	time * group	2.509	0.092	0.024
HDL(mmol/L)	Time	4.783	0.009	0.044
	Group	3.919	0.050	0.036
	time * group	1.212	0.300	0.012
LDL(mmol/L)	Time	3.010	0.052	0.031
	Group	0.120	0.730	0.001
	time * group	0.763	0.468	0.008
CASTELI	Time	1.035	0.357	0.010
	Group	1.755	0.188	0.017
	time * group	1.544	0.217	0.015
TG/TBARS	Time	1.751	0.176	0.018
	Group	2.755	0.100	0.027
	time * group	0.683	0.496	0.007
Ca	Time	101.000	<0.001	0.186
	Group	1.757	0.188	0.017
	time * group	0.091	0.763	0.001
Ca ++	Time	18.068	<0.001	0.156
	Group	0.198	0.657	0.002
	time * group	0.170	0.772	0.002

Two-way ANOVA for repeated measures. HbA1c, glycated hemoglobin; FI, fasting insulin, BMI, Body mass index; WC, waist circumference; HOMA-IR, Homeostatic Model Assessment of Insulin Resistance; FBG, fasting blood glucose; SBP, systolic blood pressure; DBP, diastolic blood pressure; MDA, malondialdehyde; AOPP, advanced oxidation protein products; CRP, C-reactive protein; TC, total cholesterol; TG, triglycerides; HDL, high-density lipoprotein; LDL, low-density lipoprotein; TBARS, thiobarbituric acid reactive substance; Ca, total calcium; Ca ++, calcium ionized.

*Interaction.

**Table 4:** Adjusted p values of measured parameters over the 6-month period of vitamin D treatment.

	3 month		6 months	
	Adjusted B (95%CI)	p-value	Adjusted B (95%CI)	p-value
Vitamin D (nmol/L)	42.25 (35.43-49.98)	<0.001	38.76 (31.72-45.80)	<0.001
HbA1c (%)	-0.20 (-0.40- -0.03)	0.050	-0.24 (-0.48- -0.006)	0.045
FI (μU/L)	-0.87 (-4.48-2.75)	0.635	-1.23 (-0.82-1.38)	0.245
BMI (kg/m2)	2.88 (-0.02-5.77)	0.052	1.73 (-1.45 – 4.89)	0.288
WC (cm)	-1.12 (-2.43-0.19)	0.094	-1.32 (-2.82-0.19)	0.086
HOMA IR	-0.70 (-2.24-0.85)	0.372	-0.61 (-1.54-0.33)	0.203
FBG (mmol/L)	-0.59 (-1.08- -0.10)	0.018	-0.38 (-0.85-0.09)	0.116
SBP (mmHg)	1.81 (-4.35-7.97)	0.561	-2.82 (-9.10-3.44)	0.373
DBP (mmHg)	0.87 (-2.63-4.38)	0.622	-1.74 (-5.05-1.57)	0.299
MDA (TBARS) (μM/L)	-0.030 (-0.60-0.54)	0.916	-0.60 (-1.55-0.94)	0.215
AOPP (µM chloramine T equivalents)	38.62 (-13.10-90.34)	0.142	5.93 (-35.42-47.28)	0.776
CRP (mg/L)	-1.40 (-6.50-3.70)	0.588	1.64 (-3.31-0.56)	0.385
TC (mmol/L)	-0.17 (-0.50-0.16)	0.321	0.06 (-0.35-0.48)	0.756
TG (mmol/L)	-0.42 (-0.76- -0.08)	0.019	-0.46 (1.08-0.16)	0.156
HDL (mmol/L)	-0.42 (-1.24-0.39)	0.305	0.04 (-0.03-0.11)	0.270
LDL (mmol/L)	-0.53 (-1.55-0.49)	0.306	0.25 (-0.14-0.64)	0.209
Castelli I	-0.40 (-0.83- -0.05)	0.040	-0.26 (-0.73-0.21)	0.409
TG/TBARS	-0.11 (-0.23-0.01)	0.078	-0.06 (-0.18-0.05)	0.288
Ca	0.18 (-0.02-0.06)	0.369	0.19 (-0.02-0.06)	0.333
Ca ++	0.00 (-0.03-0.03)	0.989	-0.005 (-0.07-0.06)	0.874

B was adjusted for baseline value of specific parameter, age, gender, FI, BMI and HbA1c. HbA1c, glycated hemoglobin; FI, fasting insulin; BMI, Body mass index; WC, waist circumference; HOMA-IR, Homeostatic Model Assessment of Insulin Resistance; FBG, fasting blood glucose; SBP, systolic blood pressure; DBP, diastolic blood pressure, MDA, malondialdehyde; AOPP, advanced oxidation protein products; CRP, C-reactive protein; TC, total cholesterol; TG, triglycerides; HDL, high-density lipoprotein; LDL, low-density lipoprotein; TBARS, thiobarbituric acid reactive substances; Ca, total calcium; Ca ++, calcium ionized.

For HbA1c, there are significant effect of group (F=9.782, p=0.037), and significant effect of time (F=9.782, p<0.001) (**Table 3**).

**Figure 2:**
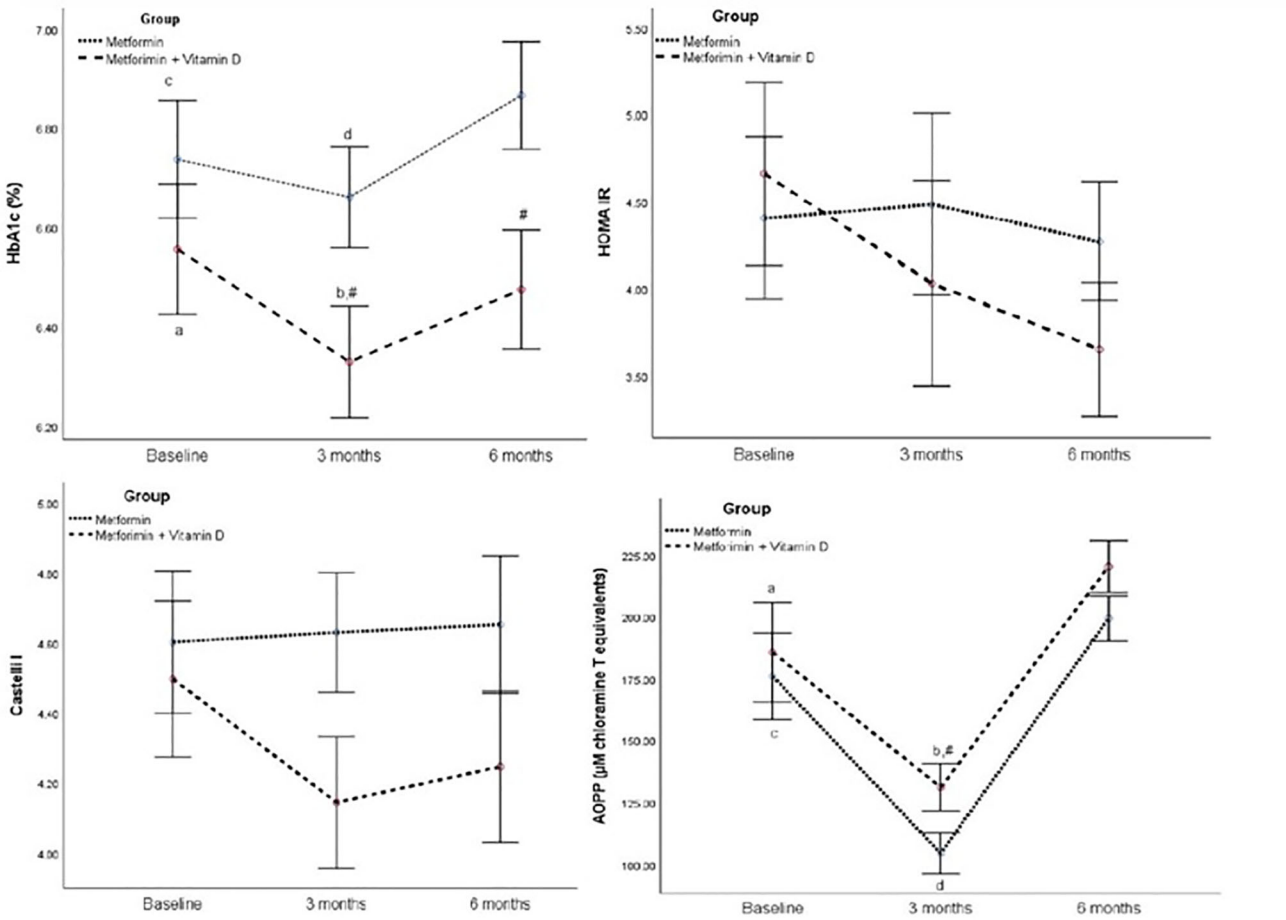
HbA1c, HOMA IR, Castelli I, and AOPP in the follow-up of 6 months between Metformin and Metformin + Vitamin D group. Data are presented mean ± SE, In two way ANOVA for repeated measures: In the Metformin + Vitamin D group: p<0.05 a vs 3 months, ^b^ vs 6 months, In the Metformin: p<0.05 ^c^ vs 3 months, ^d^ vs 6 months, # between group at specific time point.

In Metformin + Vitamin D group, HbA1c levels decreased significantly after 3 months of supplementation (p=0.011) and increased significantly between 3 and 6 month of supplementation (p<0.001). Significant difference in HbA1c levels between groups was seen after 3 (p=0.031) and after 6 months (p=0.017) (**Figure 2**).

For FBG, SBP, MDA, TC, Ca, and Ca ++ repeated measures two-way ANOVA revealed significant effect of time (p=0.020, p=0.046, p<0.001, p=0.008, p<0.001, and p<0.001) (**Table 3**), but there was no significant difference between groups during the intervention period.

For BMI, repeated measures two-way ANOVA revealed no significant interaction (F=2.175, p=0.129), significant effect of group (F=4.489, p=0.039), and significant effect of time (F=4.926, p=0.015) (**Table 3**). For DBP, repeated measures two-way ANOVA revealed no significant interaction (F=0.528, p=0.567), significant effect of group (F=4.501, p=0.039), and significant effect of time (F=3.989, p=0.029) (**Table 3**).

For AOPP, repeated measures two-way ANOVA revealed no significant interaction (F=0.098, p=0.907), significant effect of group (F=4.214, p=0.047), and significant effect of time (F=90.323, p<0.001) (**Table 3**). Significant difference between groups was seen only in 3^rd^ month of intervention time (**Table 4**).

Regarding mean change, regression analysis significant differences between groups were seen in Vitamin D after 3 months (B 42.25 (95%CI 35.43 - 49.98), p<0.001), and after 6 months (B 38.76 (95%CI 31.72 – 45.80), p<0.001). Also, it is demonstrated a significant mean difference of 0.20 mmol/L in HbA1c after 3 months (p=0.050), of 0.24 mmol/L in HbA1c after 6 months (p=0.045).

## Discussion

In the present study we have found that the recommended doses of vitamin D significantly decreased the level of HbA1c after 3 as well as after 6 months of vitamin D supplementation in patients with T2DM treated with metformin, compared to the metformin group. The most recent meta-analysis carried out by Hu at al. indicated that vitamin D supplementation had beneficial effects on HbA1c, insulin resistance and FI in the subgroup of subjects with short-term follow-up intervention, but had no effect among those with more than 3 months of intervention (20). Similar results were obtained in the systematic review by Nigil Haroon et al. (21). There are many causes which could support these results. First of all, it should not be ignored that the 6-month period is the time of three seasonal changes, in our case, from summer to winter, when the climate conditions, the amount of clouds and a smaller extent of UV exposure may have a significant influence on the endogenous production of vitamin D. Furthermore, the diet and daily habits may differ significantly in winter, which may contribute to the worsening of the metabolic control. Additionally, the patient’s perception of treatment and motivation may have a significant influence on the treatment efficacy. To our knowledge, this is the first study with individual dosing plan for patients regarding 25(OH)D levels according to the Endocrine Society guidelines (11). The daily doses proposed were 7142 IU (50 000 IU weekly) for vitamin D deficient patients and 2000 IU for patients who had 25(OH)D levels > 50 nmol/L. This dose of 2000 IU was not sufficient to provide 25(OH)D levels beyond 100 nmol/L, which is believed to be crucial for extra-skeletal effects (22). The same fact could explain why we did not maintain a significant effect of vitamin D on FI and HOMA-IR levels over the six-month period. Talaei et al. showed that the effect of vitamin D on insulin resistance was significant only when the vitamin D concentration was between 100 and 150 nmol/L (22). Since the definition of the optimal vitamin D level remains controversial, vitamin D deficiency would not be the inclusion and standard judgment criterion (23, 24). However, it is possible that vitamin D has beneficial effects only in vitamin D deficient patients especially in those with poor glycaemic control (25, 26). Yet, Krul-Poel et al. found that vitamin D supplementation was not sufficient to improve glycaemic control in patients with well controlled T2DM, but they used intermittent high-dose of vitamin D. They may have an impact on different outcomes compared to daily doses (27, 28).

A meta-analysis that included 17 randomized controlled trials evaluated the effect of vitamin D supplementation on lipid profile in patients with T2DM. Results revealed that vitamin D supplementation had positive effects in vitamin D deficient patients who had received vitamin D intervention for ≤ 12 weeks. Our results also showed that vitamin D had beneficial effect on HDL cholesterol after 3 months of supplementation when there was higher prevalence of vitamin D deficiency in the Vitamin D+Metformin group and higher vitamin D doses were given (29).

The proposed mechanisms of vitamin D beneficial effects may occur on different levels. By acting as an immunomodulator, vitamin D can regulate innate and adaptive immunity, particularly dendritic cell maturation, macrophage differentiation and T-cell proliferation, while decreasing inflammatory response, inflammatory cytokine secretion and apoptosis of beta cells. On the cellular level, it can down-regulate inflammatory and redox sensitive nuclear transcription factor kappa beta (NFκB) (30–33). Vitamin D stimulates insulin secretion by calcium flux-dependent mechanisms (34).

Although we observed decrease in CRP levels in Metformin + Vitamin D group this decrease did not reach statistical significance. The lack of significant difference between groups in our study could be explained by a small sample size.

We also observed significant increase in Ca and Ca ++ levels in both groups during the study period (p<0.0001). Still, there was no significant difference between groups in Ca and Ca++ levels. Seasonal changes in sunlight and dietary habits in spite of given recommendations could be the reason for this result.

Oxidative stress is strongly associated with T2DM and plays a key role in insulin peripheral sensitivity and insulin secretory response (6, 35). It is well documented that hyperglycaemia contributes mostly to the generation of the reactive oxygen species (ROS), through glucose autoxidation and consequent non-enzymatic glycation of proteins in T2DM (36, 37). Recent evidence has suggested that Vitamin D can decrease oxidative stress. Meta-analysis performed by Sepidarkish et al. has shown that vitamin D supplementation significantly reduced levels of MDA, a primary biomarker of lipid peroxidation (38, 39). In the same meta-analysis the positive effect on MDA was observed only in subgroups with bi-weekly administration of vitamin D doses that are between 100 000 and 200 000 IU per month. Doses less than 100 000 IU and more than 200 000 IU per month were under significant effect. Different dosing and different way of administration could be the reason for not having more pronounced effects of vitamin D on MDA levels during our study. Similar results were reported by Eftekhari et al. (40). It is well documented that vitamin D possess the antioxidant potential *in vivo* and *in vitro*, serving as a lipophilic substance, as a membrane and lipoprotein free radical scavenger and antioxidant (41–43). It seems that vitamin D treatment was not a promising approach in improving the AOPP level, most probably because of its lipophilic properties (**Table 2**). Beneficial effect was seen only after 3 month period when higher doses of vitamin D were given. After 3^rd^ month AOPP had the same trend of rising in both groups, which can be attributed to the interaction of drugs present in circulation (most probably metformin) with method, since all samples were made at once, under the same conditions.

Our study had some limitations. Firstly, it was a randomized but not a placebo controlled trial. Levels of 25(OH)D were used as vitamin D status markers, but vitamin D has several forms with different levels of activity (1). Insulin resistance was assessed using HOMA IR instead of hyperinsulinemic-euglycemic clamps, which is proposed as a gold standard (44). Serum 25(OH)D was measured by an electrochemiluminescence immunoassay, not a gold standard method-liquid chromatography mass spectrometry (45). The strengths of our study include its randomized, prospective design; the use of vitamin D status dependent on oral daily doses according to ES guidelines; the study was mediumd and long-term follow-up randomized.

## Conclusion

Our study has indicated that oral daily doses of vitamin D proposed by ES guidelines reduce the levels of HbA1c over a 3-month and over a 6-month period. Its effect on metabolic control, through the improvement on HOMA-IR, and oxidative stress measured through AOPP improvment might have a promising effect if vitamin D could be maintained in optimal dose. Further investigation would reconsider vitamin D doses in patients with T2DM, which may attenuate the oxidative stress risk, the risk of metabolic syndrome and the related cardiovascular events.

## Data Availability Statement

The raw data supporting the conclusions of this article will be made available by the authors, without undue reservation.

## Ethics Statement

The studies involving human participants were reviewed and approved by the Ethical Committee of Primary Health Care Center in Podgorica (ID number 05/01-E.K.-5989/1). The patients/participants provided their written informed consent to participate in this study.

## Author Contributions

MC contributed in the conception of the work, participated in the study design, and wrote the manuscript. RK contributed in the conception of the work, participated in the study design, and critically revised the manuscript. AK and GK contributed in the conception of the work, performed biochemistry analyses, and critically revised the manuscript. All authors contributed to the article and approved the submitted version.

## Funding

The work was supported by the Project TR31060 (Ministry of education, science and technological development).

## Conflict of Interest

The authors declare that the research was conducted in the absence of any commercial or financial relationships that could be construed as a potential conflict of interest.

## Publisher’s Note

All claims expressed in this article are solely those of the authors and do not necessarily represent those of their affiliated organizations, or those of the publisher, the editors and the reviewers. Any product that may be evaluated in this article, or claim that may be made by its manufacturer, is not guaranteed or endorsed by the publisher.
